# Implementing health care reform: implications for performance of public hospitals in central Ethiopia

**DOI:** 10.7189/jogh.08.010403

**Published:** 2018-06

**Authors:** Tsegahun Manyazewal, Mokgadi C Matlakala

**Affiliations:** 1 Department of Health Studies, College of Human Sciences, University of South Africa, Pretoria, South Africa; 2 University of California San Diego, Anti-Viral Research Center, ADDIS VP Project, Addis Ababa, Ethiopia

## Abstract

**Background:**

Understanding the way health care reforms have succeeded or failed thus far would help policy makers cater continued reform efforts in the future and provides insight into possible levels of improvement in the health care system. This work aims to assess and describe the implications of health care reform on the performance of public hospitals in central Ethiopia.

**Methods:**

A facility-based, cross-sectional study was carried out in five public hospitals with different operational characteristics that have been implementing health care reform in central Ethiopia. The reform documents were reviewed to assess the nature and targets of the reform for interpretive analysis. Adopting dimensions of health system performance as the theoretical framework, a self-administered questionnaire was developed. Consenting health care professionals who have been involved in the reform from inception to implementation filled the questionnaire. Cronbach’s alpha was measured to ensure internal consistency of the instrument. Descriptive statistics, weighted median score, χ^2^, and Mann-Whitney U and Kruskal-Wallis tests were used for data analysis.

**Result:**

s Despite implementation of the reform, the health care system in public hospitals was still fragmented as confirmed by 50% of respondents. Limited effects were reported in favour of quality (48%), access (50%), efficiency (51%), sustainability (53%), and equity (61%) of care, while poor effects were reported in patient-provider (41%) and provider-management (32%) interactions. Though there was substantial gain in infrastructure and workspace, stewardship of health care resources was less benefited. The predominant hindrances of the reform were the working environment (adjusted Odds Ratio (aOR) = 2.27, 95% confidence interval (CI): 1.15-4.47), financial resources (aOR = 3.54, 95%CI = 1.97-6.33), management (aOR = 2.27, 95% CI = 1.15-4.47), and information technology system (aOR = 3.15, 95% CI = 1.57-6.32).

**Conclusion:**

s The Ethiopian health care reform has laid the groundwork for health system improvement, but progress was slow and the health care delivery system was still fragile. Healthcare reform efforts in such settings are feasible, but with regular mapping of programmatic outcomes and bringing a common understanding of the reform among stakeholders.

In the wave of the pressure combating health care challenges and visioning quality of care, the Ethiopian government urged a countrywide health care reform initiative in the form of Business Process Reengineering (BPR). Tracking clients’ allegations about quality of health care, the initiative imposed the Ethiopian Federal Ministry of Health (FMoH) transform the country’s health care system [1]. Rooted on BPR principles [2], the FMoH conducted “as is” analysis to capture credible evidences of the existing health care system and realize the different dynamics that should be considered in the redesign of the new reform. With these, the “to be” business processes were designed, public health sector standards formulated, and standard operating procedures and implementation tools developed [3]. The reform was progressively implemented through a series of training sessions for managers and technicians at all levels followed by changes in staff deployment, specific job assignments and the recruitment of new staff. Stretched objectives were synthesized and sub-processes that form the core process sorted out. Public hospitals’ services were re-structured into three major case teams, namely; Emergency, Outpatient, and Inpatient; where Outpatient and Inpatient case teams were further classified into eight and nine case teams, respectively [4]. Gradually, Ethiopia implemented the BPR in all government sectors to solve the problems of hierarchical bureaucracy.

Beyond the health care reform in Ethiopia, there have been debates on implications of health care reform in resource-limited countries. In Uganda, while official reports developed by donor funded expatriate staff have tended to show a positive picture of the Ugandan health sector reform [5], other studies indicate that despite these reforms, the Ugandan health sector remains challenged by under-funding [6] and poor quality of care [7]. Kenya has been in the process of implementing health care reforms to secure a fundamental change in the functioning and performance of its health care services. Despite these efforts, the health system in Kenya is inequitable and the health financing is fragmented, requiring a systematic approach to health financing reforms to ensure health coverage and equity [8] and to reinforce Health Sector Costing Model to reach the target of the country’s Vision 2030 [9]. In Tanzanian, the government’s Decentralization by Devolution health reform deemed to improve the delivery of public health services has gained successes and faced challenges [10]. The approach benefited from the increased accountability of health workers and reduced bureaucratic procedures in decision-making, however, it was challenged by funding constraints, unnecessary political interference, lack of sufficient and technically qualified personnel, and weak supportive supervision activities [10]. In South Africa and Zimbabwe, the attempts for health care reform end with ambiguity; as key aspects of the proposed National Health Insurance (NHI) scheme remains unclear and mandatory national health insurance has been discussed for decades without a system implemented in the two countries respectively [11]. In South Africa, funding was not the central problem of the public health system; but rather the enormous inefficiencies in management and low productivity, with an urgent need to re-engineer the way health facilities are internally organized to achieve better productivity and responsiveness [12].

Similar to countries in Africa, many countries’ governments in other regions have been implementing health care reforms to review their health care systems and health care services financing and delivering approaches. China has recently been compelled to undertake health sector reforms in response to inequitably distributed health services, but tackling high medical costs has not been fruitful [13,14] and sustaining positive gains has been difficult [15]. Healthcare reform in China may succeed if vertical monitoring of the quality, equity, efficiency and effectiveness of the health sector is improved [16,17]. Brazil and Colombia have implemented extensive health care reforms for decades with the major goal of improving access, increasing efficiency and reducing health inequities, but in neither case have reforms seemed to have had a decisive positive impact on the health outcomes, instead, the countries’ health improvement decelerates in the years following the reforms [18]. During the financial crisis in European countries, health care facilities have become a focal point for health care reform strategies, which consist of reducing cost as a short-term strategy and improving performance in the long run, but the reforms emphasize cost containment measures rather than embarking on structural redesign of the health care sector [19].

Analysis of literatures reviewed reveals that health care reform efforts in Africa have limited implications on the overall health system improvements, which was mainly due to minimum commitments the countries had exerted in the implementation of the reforms. The effects of the reforms were shown to be highly influenced by political principles and the unique health concerns of each country.

Healthcare institutions intend to use different business models such as total quality management, continuous quality improvement, just-in-time, BPR, benchmarking and among others to re-design their processes [20]. BPR, which is the focus of this study, is the fundamental rethinking and radical redesign of business processes to achieve improvements in critical, contemporary measures of performance, such as cost, quality, service, and speed to achieve substantial gains in the overall organizational performance [2]. It relies upon questioning, challenging, evaluating, and redesigning every element of an institution’s operational process [21]. BPR project requires specific steps to succeed to a positive outcome. The process begins with defining the scope and objectives of the reengineering project, followed by evaluating the existing (As-Is) performance of the organization and the commitment of senior management to implement the project [22,23]. The later step involves development of executive consensus on the need of the BPR, identifying the strengths and weaknesses of the organization, identifying the processes to be reengineered, defining the ultimate benefit, and clarifying that is needed and what will be their role [24]. The third step is designing the new (To-Be) process based on the data obtained from the As-Is mapping, thereby implementing the To-Be process. The implementation phase requires initiating culture change program, developing a transition plan and piloting the new process, providing training for the implementers, and executing the new process in full scale [25,26]. Once BPR is implemented, it requires monitoring the progress of action by measuring how much more informed the people feel, how much more commitment the management shows and how well the new teams are accepted in the broader perspective of the organization [27].

Although the present study is limited to Ethiopia, there are studies in other countries which reported a dramatic improvement of health care services due to implementation of health care reform using BPR as a tool. For instance, in Singapore, the second largest hospital in the country was able to eliminate the waiting time of patients with BPR oriented health care reform [28]. In Italy, a study of the use of BPR in the surgical ward of a hospital was able to identify areas for improvement such as the number of operating sessions, preparation of the operating rooms for each operation, and availability of specific surgical instruments [29]. Juxtapose this, some countries failed implementing health care reforms when using BPR as a tool [30] because implementation of BPR requires adequate financial resources [31] and knowledge about BPR concepts [32] for making changes.

Though health care reform relies on a good design, its success ultimately depends on careful attention to the complex details of implementation [33]. Healthcare reform is complex as it needs high investment in facilities, technologies, sufficient supply of pharmaceuticals, training of health workers, and a system for quality improvement [34]. The gap between health care reform framework and the support made available for implementation creates pressure for policy and decision makers, managers, and health professionals [35]. Long-term political commitment of the local governments [36] and the sense of belonging of the health care team [37] are shown to affect health care reform strategies.

In Ethiopia, despite the fact that the Ethiopian government began implementing health care reform through BPR in 2009, there is limited evidence demonstrating its success or failure. Understanding the way health care reforms have succeeded or failed thus far would help policy makers cater continued reform efforts in the future and provide insight into possible levels of improvement in the health care system. Thus, this work aims to assess and describe the implications of health care reform on the performance of public hospitals in central Ethiopia.

## METHODS

### Theoretical framework

The theoretical framework of this study was the dimensions of “health system performance” [38]. This approach presents indicators for five key dimensions of health system performance which map the linkages between health sector reform, changes in health system performance, and changes in health status; namely, quality, access, equity, efficiency, and sustainability. In the same way, the current study has taken those requirements as the major criteria that should be achieved in public hospitals of Ethiopia following the implementation of the BPR health care reform. These five key dimensions and their constructs which determine the effectiveness of the reform are explained with application to the results of this study.

### Setting and participants

A facility based, cross-sectional study was conducted in quantitative methods in major and highly complex public hospitals in Addis Ababa, Ethiopia, between January to June 2015. Addis Ababa was selected among the 11 administrative divisions of Ethiopia considering its status as the largest and city capital of Ethiopia. The Addis Ababa Health Bureau has been implementing the health care reform designed in six public hospitals. The bureau administers these public hospitals, which deliver advanced preventive and curative health services, from which five have been implementing the BPR health care reform since its inception in 2009. The five hospitals were purposively included as the study sites to maximize the scope of the study thereby ensure external validity.

The study population was all health care providers that were working in the study sites at the time of data collection (n = 1681). Of this, those who were employed at least one year before the inception of the reform (n = 476, 28%) were purposively selected to include respondents who knew the performances of the hospitals before implementation of the reform and who could better analyse the changes that occurred due to the reform. The health care providers included medical doctors, laboratory professionals, nurses, health officers, pharmacists, dentists and sanitarians.

### Data collection instrument

The reform documents as well as previous health care reform initiatives in the country were reviewed to assess the nature and targets of the reform for interpretive analysis. In order to develop a quality questionnaire, we reviewed secondary data; assessed related studies conducted previously, determined the target population and their educational levels, and considered the advice of experts before designing the questionnaire. Additionally, an in-depth review of literature was conducted to identify critical factors that could influence the success of BPR programs. Finally, six BPR critical success factors were identified; namely, financial resources, top management commitment and support, training, collaborative working environment, flatter structure, and information technology [31,39-43].

A self-designed structured, close-ended questionnaire with 82-items and close-ended 5-level scale [44] was used to collect data. The layout of the questionnaire was divided in logical order into six contents of the health system performance dimensions indicated above; namely, quality, access, equity, efficiency and sustainability, along with demographics.

In the “quality” component of the questionnaire, 32 items (Cronbach’s alpha = 0.960) were included to analyse the reform’s implication on quality of health care services. The quality investigation was guided by three health care quality dimensions: structure-process-outcome, as proposed in the Donabedian quality-of-care framework [45]. We included 22 items to measure the “outcome” quality (Cronbach’s alpha = 0.958); eight items focusing on patient-provider interaction; four items on documentation, and monitoring and evaluation; and nine items on provider-management interactions constructed from the targets depicted by the Ethiopian government in its reform document [4]. For “process”, we included eight items (Cronbach’s alpha = 0.921) to assess appropriateness of the methods and procedures followed in the implementation of the reform. “Structure” had two items aimed at assessing improvements of the overall structure of the hospitals to meet the daily workflow.

“Access” was examined using 25 items (Cronbach’s alpha = 0.960) in the questionnaire; based on the five dimensions of health care access; namely, physical, economic, temporal, cultural, and approachability dimensions. The “physical” dimension assessed availability of work space, furniture, equipment, supplies, medications, reagents, communication materials, and other supplies in the hospitals after implementation of the reform. The “economic” dimension assessed the overall effect of the reform on financing and financial management system, and “temporal” dimension assessed the effect of the reform on improving turn-around-time of the hospitals’ health care services. “Cultural” dimension assessed acceptability of the hospitals’ services, and “approachability” dimension assessed the effect of the reform on improving awareness of the community that some form of health services exists, can be reached, and have an impact on their health.

The “equity” section included four items in the questionnaire to assess availability of resources and systems in the hospitals that would benefit every citizen. “Efficiency” component used twelve items to assess the technical, economic and allocative processes related to how and which services are produced in the reform process. “Sustainability” of hospitals’ services was assessed using nine relevant items in the questionnaire.

The respondents received and completed the study questionnaire in paper-based form while they were on their working area. Each of the five responses in the questionnaire had a numerical value (1-5), in which the highest two scoring answers (4 and 5) were perceived as positive response answers, the lowest two scoring answers (1 and 2) were considered negative response answers, and the middle response answer (3) was perceived neutral. As the questions’ items were grouped into health care performance dimensions, a scale score was computed as the mean of the scales’ item scores.

The six BPR critical success factors identified in our literature review were used as a guide in identifying and analysing the factors that influence implementation of the BPR health care reform. From the study’s questionnaire, the item stating “the hospital becomes a better treatment facility” was taken as the outcome variable to indicate whether there was hospital service improvement. This item had original responses classified in five level scale. The responses were dichotomized into “Good” or “Poor” answers by taking the “Strongly agree” and “Agree” responses as a “Good” value while “Strongly disagree”, “Disagree”, and “Neutral” as “Poor” value for feasibility of analysis and interpretation. The six BPR critical success factors were taken as the explanatory variables. For each of the six success factors, three items which best describe the factor were pooled from the questionnaire and the responses given to the items analysed as a cumulative effect. Responses were valued as “Good” if at least two of the three items had a “Strongly agree” or “Agree” response in the original scale questionnaire, while the remaining responses were taken as “Poor” value. Associations of health service improvement with the six explanatory variables were tested independently using bivariate analysis. Based on the results, the independent variables were selected for the logistic regression analysis. Subsequently, multivariate logistic regression analysis was conducted to exclude confounders.

Reliability of the data collection instruments was ensured by running Cronbach's alpha test [46] for each category of items, and the results were found satisfactory. The questionnaire included close-ended questions to ensure a rational reproducibility of the study. The questionnaire was pre-tested at the study sites to ensure reliability of the instrument. Internal validity was increased through reviewing and analysing previous questionnaires and crosschecking the collected data. The inclusion of all five public hospitals that participated in the reform from inception as the study sites maximized the scope of the study to the target population. We emphatically believe that public health care providers, who are the ultimate resources of health systems, were the best candidates to validate the success or failure rate of the BPR health care reform implemented in Ethiopia. We employed the perceptions of health care providers to assess and describe the implications of the health care reform due to multiple reasons. The exclusion of patients was based on the fact that there are unwanted variations in health care practice and outcomes that cannot be explained by patients [47] and health care improvements resulting from patients’ feedback is limited [48], while providers feedback and involvement is critical as providers can realize the overall implications of health care reforms [49-52]. We also believe that the major reform the Ethiopian government designed and implemented to date to enhance the health care service delivery system in the country is the BPR health care reform. In this manner, this reform, which we assessed, is responsible for major public health care services’ gains or losses.

The study was granted ethical clearance from the Higher Degrees Committee of the Department of Health Studies, University of South Africa and the Research and Technology Transfer Core-process of the Addis Ababa City Administration Health Bureau. Written informed consents were obtained from each respondent before completing the questionnaire.

Data analysis was done through calculation of several statistical procedures on IBM SPSS version 20 (IBM Corp, Armonk, NY, USA) and on Excel 2010 (Microsoft Corp, Seattle, WA, USA). The variables were re-coded and dichotomized where appropriate on SPSS. Descriptive statistical analysis [46] was conducted to describe the means, standard deviations, medians and frequencies of items aimed at measuring quality, access, equity, efficiency, and sustainability of health care services. The weighted median scores [53] were used to demark cut-off points and categorize the perceived health service improvements. Non-parametric analysis, namely Mann-Whitney test and Kruskal-Wallis test [54], were conducted to statistically test if there was a significant difference in answering tendencies of respondents with different groups. χ^2^ test [46] was used to evaluate association of different variables, and *P* < 0.05 at 95% CI was taken as mark for statistical significance. The association between health service improvement and BPR critical success factors were tested independently using bivariate analysis. Variables with significant associations were analysed further with logistic regression analysis [55].

## RESULTS

### Socio-demographic profiles

Of the total participants who were eligible as they have worked in the hospitals for at least a year before the inception of the reform (n = 476), the questionnaire was distributed to those who consented (n = 465). The questionnaires returned (n = 410, 88%) were rechecked for completeness and those completed (n = 406) presented for analysis. Majority of the respondents were nurses (n = 304, 74.9%) followed by medical doctors (n = 35, 8.6%), and medical laboratory professionals (n = 24, 5.9%). The demographic profiles are represented in detail in **Table 1****.**

**Table 1:** Socio-demographic characteristics of respondents

Item	Count (n = 406)	%	Cumulative %
**Gender:**			
Male	124	30.5	30.5
Female	282	69.5	100.0
**Age (years):**			
20-29	93	22.9	22.9
30-39	195	48.0	70.9
40-49	92	22.7	93.6
50-59	26	6.4	100.0
**Duration of work as health professional (years):**			
6-9	146	36.0	36.0
10-19	202	49.8	85.7
20-29	55	13.5	99.3
30-39	03	0.7	100.0
**Duration of work as staff in this hospital (years):**			
6-9	247	60.8	60.8
10-19	136	33.5	94.3
20-29	21	5.2	99.5
30-39	02	0.5	100.0
**Profession:**			
Medical Doctor	35	8.6	8.6
Laboratory	24	5.9	14.5
Pharmacy	16	3.9	18.5
Nurse	304	74.9	93.3
Health Officer	14	3.4	96.8
x-ray technician	11	2.7	99.5
Sanitarian	2	0.5	100.0
**Level of education:**			
Certificate	2	0.5	0.5
Diploma	37	9.1	9.6
Degree	342	84.2	93.8
MSc/MA or MPH	7	1.7	95.6
Medical Doctor Degree + Specialty	18	4.4	100.0
**Total**	**406**	**100**	**100**

### Quality

Quality was explained by three dimensions. For the outcome, 42% responses indicated that the reform did not meet the perceived patients-provider interaction. The lowest score in patient-provider interaction was for bed appointments, where 341 (84%) of respondents claimed that the perceived time limit of 10 minutes allotted in the reform document for patients getting beds has not been met (**Table 2**). For documentation, 102 (25.1%) respondents agreed that the reform allowed reporting systems of the hospitals to be easy and time-efficient. Additionally, 130 (32%) respondents agreed that hospital guidelines and protocols are up to date and appropriate. For providers-hospital management interaction, respondents’ feedback showed that the hospital staff were not promoted to a relatively higher position (n = 246, 60.6%) or got recognition of their outstanding performance (n = 255, 62.8%). Besides, the reform did not allow for increases in salaries of staff (n = 256, 63.1%) nor did it create a better feeling of overall job satisfaction among staff (n = 228, 56.2%). The relatively positive input of the reform was on work relationship, in that the reform created better working relationship among staff (n = 204, 50.2).

**Table 2:** Descriptive analysis of health care quality

Perceived service quality	Agree frequency (%)	Neutral frequency (%)	Disagree frequency (%)
**Outcome**			
**Due to the BPR health care reform:**			
1.1.1. Out patients are completing treatment services within 2 hours	144 (35.5)	42 (10.3)	220 (54.2)
1.1.2. Emergency patients are receiving treatment immediately	232 (57.1)	67 (16.5)	107 (26.4)
1.1.3. Patients are getting beds within 10 min	27 (6.7)	38 (9.4)	341 (84)
1.1.4. Patients are receiving specialized services within 72 hours	199 (49)	63 (15.5)	144 (35.5)
1.1.5. Customers are receiving medical certificate within 1 hour	237 (58.4)	85 (20.9)	84 (20.7)
1.1.6. Patients satisfied with the hospital services	184 (45.3)	114 (28.1)	108 (26.6)
1.1.7. Treatment & respect of patients improved	213 (52.5)	79 (19.5)	114 (28.1)
1.1.8. Missing patients’ medical records is rare	107 (26.4)	66 (16.3)	233 (57.4)
**% Patient-provider interaction**	41%	17%	42%
1.1.9. Reporting systems are easy and not time consuming	102 (25.1)	76 (18.7)	228 (56.2)
1.1.10. Guidelines & protocols are up-to-date & appropriate	130 (32)	87 (21.4)	189 (46.6)
1.1.11. Opportunities to learn from successes/challenges created	190 (29.6)	96 (23.6)	120 (46.8)
1.1.12. Up-to-date technologies for patient diagnosis are in use	150 (36.9)	92 (22.7)	164 (40.4)
1.1.13. Monitoring & evaluation systems are established	181 (44.6)	94 (23.2)	131 (32.3)
**% Documentation & progress monitoring**	37%	22%	41%
1.1.14. Staff developed good working relationship with each other	204 (50.2)	82 (20.2)	120 (29.6)
1.1.15. Staff receive appropriate & timely performance feedback	131 (32.3)	78 (19.2)	197 (48.5)
1.1.16. Staff have clear job description	188 (46.3)	96 (23.6)	122 (30)
1.1.17. Staff get a better feeling of overall job satisfaction	111 (27.3)	67 (16.5)	228 (56.2)
1.1.18. Staff are highly motivated to their work	96 (57.4)	77 (19)	233 (23.6)
1.1.19. Staff salary increases	93 (22.9)	57 (14)	256 (63.1)
1.1.20. Staff promoted to a relatively higher position	92 (22.7)	68 (16.7)	246 (60.6)
1.1.21. Staff with outstanding performance are getting recognition	93 (22.9)	58 (14.3)	255 (62.8)
1.1.22. Staff use their working hours appropriately	166 (40.9)	59 (14.5)	181 (44.6)
**% Staff-hospital management system interaction:**	32%	18%	50%
**% Outcome**	37%	18%	45%
**Process:**			
**In the BPR implementation process:**			
1.2.1. Staff are able to clearly know the mission and vision of the hospital	218 (53.7)	130 (32)	58 (14.3)
1.2.2. Supervisors were coming from health bureau to monitor the reform effort	119 (29.3)	156 (38.4)	131 (32.3)
1.2.3. Hospital quality improvement goals were known throughout case teams	199 (49)	109 (26.8)	98 (24.1)
1.2.4. Hospital employees were involved in developing plans	165 (40.6)	78 (19.2)	163 (40.1)
1.2.5. Adequate training has been provided to all staff	109 (26.8)	51 (12.6)	246 (60.6)
1.2.6. Stakeholders communicated on the new design and feedbacks received	68 (16.7)	144 (35.5)	194 (47.8)
1.2.7. Feedbacks from patients and data from pilot test were incorporated	90 (22.2)	114 (28.1)	202 (49.8)
1.2.8. The right team members have been prepared to process the reform	117 (28.8)	123 (30.3)	166 (40.9)
**% Process:**	33%	28%	39%
**Structure:**			
**Due to implementation of BPR health care reform:**			
1.3.1. The way the hospital is structured is conducive to the daily work flow	161 (39.7)	110 (27.1)	135 (33.3)
1.3.2. The hospital becomes a better treatment facility	202 (49.8)	136 (33.5)	68 (16.7)
**% Structure**	45%	30%	25%

BPR – business process reengineering

The overall analysis of findings indicates that provider-management system interaction of the hospitals is still weak. The weighted median descriptive statistics made on outcomes of quality from visual binning of the variables, suggest that 207 (51%) of the respondents argued that the BPR health care reform has brought improvements on hospital quality outcomes, while 199 (49%) indicated that there are no improvements. For process, among the major gaps in the health care reform implementation process was training, and 246 (60.6%) of the respondents explained that adequate training had not been provided to all staff throughout the BPR implementation process. Additionally, only 68 (16.7%) of respondents agreed that the reform process involved stakeholders on the new design and received their feedback (**Table 2**). Overall, 211 (52%) of respondents indicated that appropriate procedures have not been followed in the processing of the reform. For structure, 52.5% of respondents argued that there is no improvement in the structure of the hospitals, while 322 (65%) of participants agreed that the hospitals became conducive to the daily workflow.

From the analysis of the weighted median score from visual binning of the variables, 211 (52%) of respondents indicated that there are no improvements in quality of health care services due to implementation of the BPR health care reform.

### Access

Access was explained by five dimensions, with which, 206 (50.7%) of respondents indicated that the reform enabled the hospitals organized with case teams that have well-defined rooms or spaces adequate to the daily work flow (**Table 3**). However, according to the respondents, there were other physical barriers which were compromising their day-to-day activities. The respondents claimed that after implementation of the reform, the hospitals still have insufficient office furniture (n = 227, 55.9%), stationery materials (n = 238 58.6%), and reagents and drugs (n = 215 53%). Additionally, the hospitals lack conducive staff rest rooms (n = 266 65.5%) and clean work areas (n = 192 47.3%). Implementation of the reform did not empower the hospitals to get equipped with internet services (n = 332, 81.8%), functioning computers (n = 343 56%) and a functioning landline telephone to call within and outside the hospitals (n = 239 58.9%). Preventive and curative maintenance of diagnostic equipment were also described as ineffective. The overall finding indicated that implementation of the reform did not address the shortages or absence of critical physical dimensions that are needed for the hospitals’ day-to-day services.

**Table 3:** Descriptive analysis of health care access

Perceived access	Agree frequency (%)	Neutral frequency (%)	Disagree frequency (%)
**2.1. Physical dimension**			
**After the BPR health care reform, the hospital has:**			
2.1.1. Defined room/spaces for each case team	206 (50.7)	94 (23.2)	106 (26.1)
2.1.2. Enough office furniture	140 (34.5)	39 (9.6)	227 (55.9)
2.1.3. Enough stationery materials	128 (31.5)	40 (9.9)	238 (58.6)
2.1.4. Enough reagents/drugs/supplies to perform daily activities	111 (27.3)	80 (19.7)	215 (53.0)
2.1.5. Clean work area	118 (29.1)	96 (23.6)	192 (47.3)
2.1.6. Conducive staff rest room	67 (16.5)	73 (18)	266 (65.5)
2.1.7. Functioning computers as needed	154 (37.9)	62 (15.3)	190 (46.8)
2.1.8. Internet access	46 (11.3)	28 (6.9)	332 (81.8)
2.1.9. Backup generator whenever needed	251 (61.8)	103 (25.4)	52 (12.8)
2.1.10. A functioning and accessible landline	98 (24.1)	69 (17)	239 (58.9)
2.1.11. Adequate maintenance service when a diagnostic machine fails	77 (19)	85 (20.9)	244 (60.1)
2.1.12. A scheduled equipment preventive maintenance services	39 (9.6)	107 (26.4)	260 (64)
**% Physical dimension**	**29%**	**18%**	**53%**
**2.2 Economic dimension**			
**After implementation of BPR health care reform:**			
2.2.1. Efficient and effective health care financing system has been established	141 (34.7)	184 (45.3)	81 (20)
2.2.2. Financial mobilization is linked with evidence-based plan	123 (30.3)	135 (33.3)	148 (36.5)
2.2.3. Hospital income increased	207 (51)	162 (39.9)	37 (9.1)
2.2.4. Budget consumption becomes effective	142 (35)	151 (37.2)	113 (27.8)
2.2.5. Corruption suspects decreased	144 (35.5)	97 (23.9)	165 (40.6)
**% Economic dimension**	**37%**	**36%**	**27%**
**2.3 Temporal dimension:**			
**After implementation of BPR health care reform:**			
2.3.1. Patients receive hospital services on time	223 (54.9)	81 (20)	102 (25.1)
2.3.2. Patients’ appointment wait-time is reasonable	216 (53.2)	81 (20)	109 (26.8)
2.3.3. Patients’ time spent while waiting in reception is reasonable	219 (53.9)	72 (17.7)	115 (28.3)
**% Temporal dimension**	54%	19%	27%
**2.4. Cultural dimension:**			
**After implementation of BPR health care reform:**			
2.4.1. Patients receive hospital services using languages and mode of communication suitable to them	224 (55.2)	110 (27.1)	72 (17.7)
2.4.2. There is no patient discrimination	338 (83.3)	45 (11.1)	23 (5.7)
**% Cultural dimension:**	**69%**	**19%**	**0.6%**
**2.5. Approachability dimension:**			
**After implementation of BPR health care reform:**			
2.5.1. The hospital establishes a system of advocating its services to the community	157 (38.7)	136 (33.5)	113 (27.8)
2.5.2. The community is aware of the hospital’s services	213 (52.5)	131 (32.2)	62 (15.3)
2.5.3. The community understands the value of the hospital on their health	204 (50.2)	140 (34.5)	62 (15.3)
**% Approachability dimension**	**47%**	**33%**	**20%**

BPR – business process reengineering

For the economic dimension, 141 (34.7%) of respondents agreed that efficient and effective health care financing systems are in place after implementation of the reform. However, 165 (40.6%) respondents claimed that corruption in the hospitals still exists. In general, 37% responses agreed that there were improvements in economic dimensions of the hospitals, 27% disagreed and 36% were neutral. The overall result of temporal dimension of access showed reasonable improvement, with 54% level of agreement. In the cultural dimension, 224 (55.2%) of the respondents agreed that the reform enabled patients to receive health care services using a mode of communications suitable to them. Additionally, the majority of respondents (n = 338, 83.3%) agreed that there was no patient discrimination in the hospitals since the reform was implemented. The overall analysis revealed that the reform was reasonably capable of addressing the cultural dimensions of health care performance, with only 95 (0.6%) level of disagreement. Conversely, for the approachability dimension, responses indicated that the reform did not effectively address the approachability dimensions of health care reform performance.

According to analysis of the total weighted median score of the five dimensions of health care access, 50% of the respondents revealed that implementation of the BPR health care did not bring improvements on health care access.

### Equity

Responses on equity indicated that the hospitals are giving medical services with reasonable prices. The findings confirmed that the hospitals gave free services for patients who cannot afford. A total of 284 (70%) of responses indicated that patients with different socio-economic, demographic, ethnic, and/or gender groups have equal access to the hospitals’ services (**Table 4**). Overall, 61% of respondents agreed that health care equity has improved due to implementation of BPR health care reform, while 39% disagree.

**Table 4:** Descriptive analysis of health care equity, efficiency, and sustainability

3. Perceived equity	Agree frequency (%)	Neutral frequency (%)	Disagree frequency (%)
**After implementation of BPR health care reform:**			
3.1.1. Amount of money patients pay for getting hospital services is reasonable	281 (69.2)	98 (24.1)	27 (6.7)
3.1.2. The hospital gives free services for patients who cannot afford	343 (84.5)	35 (8.6)	28 (6.9)
3.1.3. The hospital has appropriate infrastructure setup for disabled patients	115 (28.3)	43 (10.6)	248 (61.1)
3.1.4. Patients with different socio-economic, demographic, ethnic, and/or gender groups have equal access to the hospital services	284 (70)	89 (21.9)	33 (8.1)
**% Equity**	**63%**	**16%**	**21%**
**4. Perceived efficiency**			
**In the BPR health care reform implementation process:**			
4.1.1. The best use of resources is observed	175 (43.1)	128 (31.5)	103 (25.4)
4.1.2. Wastage reduced and cost-effective interventions enhanced	168 (41.4)	76 (18.7)	162 (39.9)
4.1.3. Enough and competent health care workers and administrators are in place	181 (44.6)	98 (24.1)	127 (31.3)
4.1.4. Sufficient rooms are in place	173 (42.6)	98 (24.1)	135 (33.3)
4.1.5. Enough drugs and medical supplies, medical apparatuses and equipment	146 (36)	104 (25.6)	156 (38.4)
4.1.6. The staff have adequate knowledge on BPR objectives and principles	141 (34.7)	119 (29.3)	146 (36)
4.1.7. The staff is technically competent to implement the BPR reform	141 (34.7)	117 (28.8)	148 (36.5)
4.1.8. Supervisors assigned according to the BPR reform structure are capable and qualified	141 (34.7)	83 (20.4)	182 (44.8)
4.1.9. There is a clear channel of communication at workplace	176 (43.3)	51 (12.6)	179 (44.1)
4.1.10. Top management is competence to support the BPR reform	166 (40.9)	61 (15)	179 (44.1)
4.1.11. Top management involves the technical staff in decision making	153 (37.7)	55 (13.5)	198 (48.8)
4.1.12. Hospital management facilitates job-related training to staffs when necessary	142 (35)	43 (10.6)	221 (54.4)
**% Efficiency**	**39%**	**21%**	**40%**
**5. Perceived sustainability**			
**After implementation of BPR health care reform:**			
5.1.1. The hospital management is committed to maintain the BPR reform	187 (46.1)	143 (35.2)	76 (18.7)
5.1.2. The hospital is able to continuously improve performance	199 (49)	108 (26.6)	99 (24.4)
5.1.3. The hospital acquires the required financial resources to insure sustainability	181 (44.6)	125 (30.8)	100 (24.6)
5.1.4. The hospital acquires the required qualified staff to ensure sustainability	170 (41.9)	129 (31.8)	107 (26.4)
5.1.5. The hospital networking with external partners is strengthened	122 (30.0)	163 (40.1)	121 (29.8)
5.1.6. The hospital has the capacity to assemble and manage resources	150 (36.9)	130 (32.0)	126 (31.0)
5.1.7. The hospital increases satisfaction of patients and providers with clinical or administrative services	170 (41.9)	93 (22.9)	143 (35.2)
5.1.8. community-level partnerships are maintained	129 (31.8)	152 (37.4)	125 (30.8)
5.1.9. new organizational practices and policies are sustained	118 (29.1)	165 (40.6)	123 (30.3)
**% Sustainability**	**69%**	**33%**	**28%**

BPR – business process reengineering

### Efficiency

For efficiency, 175 (43.1%) respondents agreed that the best use of economic resources was achieved in the reform implementation process, and 181 (44.6%) of respondents agree that enough and competent health care workers were available in the reform implementation process. However, 179 (44%) respondents argued that the role and capacity of the hospitals’ high-level management in the reform implementation process was insufficient (**Table 4**). In general, 207 (51%) respondents claimed that there are no improvements in efficiency of health care services due to implementation of the reform.

### Sustainability

From analysis of weighted median score, 192 (47.3%) respondents agree that the reform improved sustainability, while 214 (52.7%) of respondents disagree that the reform improved sustainability of the public hospitals (**Table 4**). For sustainability, based on the overall weighted median score, 203 (50%) respondents agree that the implemented BPR health care reform was not effective in improving the health care system performance of public hospitals. A relatively higher level of agreement (n = 199, 49%) was on the hospitals ability to continuously improve performance, while the rest of responses for this category of items were neutral.

### Overall health care system

Based on the overall weighted median score result, 203 (50%) respondents claimed that the implemented BPR health care reform was not effective in improving the health care system performance of public hospitals. Analysis with the Kruskal-Wallis test indicates that there were difference in scoring tendency between respondents with different health profession (*P =* 0.001) and respondents with different duration of work in the hospitals (*P* = 0.026). Meanwhile, there were no differences in the scoring tendency between respondents with different levels of education (*P* = 0.539) and between respondents with different age groups (*P* = 0.235).

### Predictors

With the backward stepwise logistic regression analysis, the important predictors that influenced implementation of the reform were financial resources (aOR = 3.54, 95%CI = 1.97-6.33), top management commitment and support (aOR = 2.27, 95%CI = 1.15-4.47), collaborative working environment (aOR = 1.77, 95% CI = 1.00-3.11), and information technology (aOR = 3.15, 95% CI = 1.57-6.32) (**Table 5**).

**Table 5:** Logistic regression analyses of the relative effect of BPR critical success factors on health service improvement

Healthcare services							
**Factors**	**Frequency**			**df**	**Significance**	**Crude OR (95% CI)**	**Adjusted OR (95% CI)**
	**Improved**		**Not improved**				
**Adequate financial resources**	Poor	145	35	1	<0.001	11.72 (7.30-18.83)	3.54 (1.97-6.33)
	Good	59	167				
**Top management commitment & support**	Poor	162	49	1	0.018	12.04 (7.55-19.22)	2.27 (1.15-4.47)
	Good	42	153				
**Collaborative working environment**	Poor	155	72	1	0.050	5.71 (3.71-8.79)	1.77 (1.00-3.11)
	Good	49	130				
**Flatter structure**	Poor	169	63	1	0.092	10.65 (6.66-17.05)	1.80 (0.91-3.55)
	Good	35	139				
**Information technology**	Poor	187	90	1	0.001	13.70 (7.75-24.18)	3.15 (1.57-6.32)
	Good	17	112				
**Training**	Poor	175	73	1	0.218	10.66 (6.56-17.35)	*
	**Good**	**29**	**129**				

BPR – business process reengineering, CI – confidence interval, OR – odds ratio, df – degrees of freedom

*aOR not calculated as the variable had insignificant association in the bivariate analysis.

## DISCUSSION

Healthcare services that are partly supported by the government were established in Ethiopia in the late 19^th^ century. The health policy of the transitional government of Ethiopia that was approved in 1993 perceived the development of an acceptable standard of health service system as a critical element within the general health policy. Lately, based on this health policy, BPR health care reform rose and endeavoured to satisfy health care quality needs of the government and citizens. It was yet to be assessed for its implications and opportunities to improve.

The findings of this study proved that the implemented BPR health care reform struggles to meet health care quality requisites. The majority of the anticipated patient-provider interactions delineated in the reform document have not been achieved. According to the reform guideline document, emergency patients coming to public hospitals at any time ought to get the required services without any delay, whereas outpatients ought to complete treatment services within two hours and patients’ admissions should be carried out within 10 minutes [4]. Similarly, patients who require specialized health care services need to get the services within 72 hours of visiting the hospitals. The reform document conjointly urged for the satisfaction of patients with health care services and their rights fully respected. Though we did not calculate the turn-around-time of care for each patient, we boldly deduced from responses of the health care providers that there exists a high gap in meeting patients’ needs. Prior to designing and implementing the reform, the Ethiopian government was able to conduct SWOT analysis and identify major gaps that would impact patient-provider interactions, and amongst this was longer patients waiting time [4], and this study revealed that the challenge has not been addressed. Recent studies conducted in Ethiopia also reported critical gap in patient’s waiting time and patient-provider interactions in public health care facilities where the studies were conducted [56,57]. A patient-provider trust would generate smooth interaction between the two parties and further insists the community decide to engage in prevention and control interventions in the general health care systems [58-60].

The implication of the BPR health care reform was found to be poor in improving the interaction between providers and the hospital management. Appropriate and timely feedback to staff, clear job descriptions, motivation, job satisfaction, and staff promotion were not improved as providers anticipated. The finding also concurs with previous studies done in Ethiopia [61-63]. Poor provider-management interaction in public hospitals would compromise the maximum commitment and engagement providers could exert to their duties. This in turn leads the hospitals not to function to the best of their abilities. It is crucial to actually listen and respond to providers’ needs to ensure high levels of engagement throughout the hospitals. If there are factors which are beyond the control of providers, the possibilities that they become client-oriented, productive, skilled, and competent to perform their duties could lessen [6].

There were severe gaps in the implementation process of the health care reform which contributed negatively to service quality. For instance, high level supervisors had given the implementation process little attention, and this actually contradicts with BPR reform standards. The management’s support and involvement in all phases of a reform is essential for lifting technical competency of staffs and enhancing outcomes [32,50,64].

In terms of structure, the reform could not enable the hospital to become a better treatment facility than it was before, though there were some recognized improvements. As well, the way the hospitals were re-structured did not match the daily work flow of the hospitals as anticipated. In general, there have been many service quality gaps identified in the public hospitals that implemented health care reform, though there were a few improvements as well. The findings were in line with previous findings from other studies done in Ethiopia [6,58,65] and elsewhere [66,67].

Access to health care services is the other dimension of health system performance that the reform ought to address. In view of the physical dimension, the reform was able to organize the hospitals into three core case teams; namely, emergency, outpatient, and inpatient. The outpatient core case team consisted of eight activity-specific case teams, and inpatient core case team consisted of nine case teams. Though the case teams existed in the hospitals, those with major furniture, supplies, and infrastructure were considered a major implementation challenge of the health care reform as providers indicated. This finding supports other studies conducted in Ethiopia [65,68]. Financial management systems raised another key concern in the reengineered public hospitals. The result indicated that the reform was able to increase income of the hospitals to some extent. However, the hospitals’ health care financing system remained weak. Financial mobilization schemes of the hospitals did not centre on evidence-based plans and their budget consumption system was stagnant, which was in support of previous studies in Ethiopia [57,69,70]. Some hospitals that implemented the reform are suspected of corruption, and this should be further investigated by the authorized government institution. For the cultural dimension of health care access, it was indicated that patient discrimination in the hospitals highly decreased since the reform was implemented. With this, the BPR health care reform was capable of addressing the cultural dimensions of health care reform performance. In general, although the reform was capable of producing meaningful changes in the cultural dimension and fairly in the approachability dimension of health service systems, it was not able to achieve the overall access to health care services.

Healthcare equity was studied to analyse the availability of adequate resources and systems in the hospitals, which would fairly benefit every citizen. The findings of the study indicate that medical care costs of the hospitals were reasonable. The reform enabled financial procedures of the hospitals to endorse free services to patients who could not afford them. The reform also allows patients with different socio-economic, demographic, ethnic, and/or gender groups to have equal access to the hospitals’ services. However, the reform was not able to address equity for disabled patients and there has been limited infrastructure setup for disabled patients. The overall findings of the study show that the implemented BPR health care reform was relatively fair at meeting the depicted health equity needs.

Efficiency was the other dimension of health system performance assessed in this study. The effect of the BPR health care reform on improving efficiency of public hospital services was shown to be unsatisfactory. While enough and competent health care workers were not in place to efficiently implement the reform efforts, efforts exerted by the hospitals’ high level management in the reform implementation process were insufficient. It was also noticed that management did not enrich knowledge and technical competency of the staff to enable them to efficiently implement the reform. These challenges, together with high wastage and inefficient use of resources, led the reform to have a little effect on efficiency. Similarly, the reform did not ensure sustainability of public hospitals’ services to continue functioning and initiate changes in order to continuously improve performance. The commitment of hospitals’ managements to maintain the BPR reform, readiness of financial resources and qualified staff, existing network of the hospitals with external partners, and satisfaction of patients and providers with existing hospitals’ clinical and administrative functions were revealed as very weak to ensure sustainability of the hospital services.

In summary, the health care reform implemented in public hospitals of Ethiopia did not improve quality, access, efficiency, and sustainability of health services, while a relatively fair improvement was shown on equity.

This study has some limitations. The study did not solicit the views of administrative staff and we think their inputs may have further strengthened the study findings, while the possible effect of this has been mitigated by inclusion of health care providers who also have administrative role. The study bases the perspectives of the respondents that may increase the likelihood of recall biases. Despite these, the study generates important findings in the area of health care reform.

## CONCLUSIONS

It was acceptable that the Ethiopian government made its level to improve the quality of care through BPR health care reform. The reform has laid the groundwork for health system improvement but progress was slow and the health care delivery system was still fragmented. Healthcare reform efforts in such settings are feasible, but with regular mapping of programmatic outcomes and bringing a common understanding of the reform among stakeholders. Such efforts in resource-limited settings need concrete follow-up and supports consistent with national and global needs. Local governments should strengthen collaborations with global health partners for empirical interventions against the major gaps detracting their health care system.
